# Female and male partner perspectives on placebo Multipurpose Prevention Technologies (MPTs) used by women in the TRIO study in South Africa and Kenya

**DOI:** 10.1371/journal.pone.0265303

**Published:** 2022-05-12

**Authors:** Laura Danielle Wagner, Alexandra M. Minnis, Jaclyn Shea, Kawango Agot, Khatija Ahmed, Ariane van der Straten

**Affiliations:** 1 Women’s Global Health Imperative, RTI International, Berkeley, California, United States of America; 2 Youth + Tech + Health, Oakland, California, United States of America; 3 Impact Research and Development Organization, Kisumu, Kenya; 4 Setshaba Research Centre, Soshanguve, South Africa; 5 ASTRA Consulting, Berkeley, California, United States of America; Makerere University, School of Public Health, UGANDA

## Abstract

**Background:**

Male partner awareness and acceptance of microbicide and family planning product use has been shown to increase women’s own acceptance and adherence of a product. However, little is known about preferences regarding potential Multipurpose Prevention Technology (MPT) product delivery forms. As part of the TRIO study, men’s reactions to their female partner’s TRIO product use and comparisons of men’s and women’s views of TRIO product attributes and use acceptability were explored to better understand product preferences.

**Methods:**

Women in TRIO used three placebo products that represented potential MPTs: daily oral tablets, monthly vaginal rings, and monthly dual injections. Male partners (N = 39) and women (N = 88) completed in-depth interviews on their own and their partner’s experiences with these products. Qualitative coding and analyses followed a conceptual model of HIV prevention product acceptability, and here, we explored themes of disclosure, trust and infidelity as they informed barriers and facilitators to product use.

**Results:**

Men expressed a desire to know of their partner’s product use decisions and be informed and educated on the products to better support their partners, in some cases, expressing a high level of concern regarding maximizing the ease of product adherence for their partner. They also wanted to understand the effects of products on sexual encounters with their partner, but in some cases, wanted more knowledge in order to control their partner’s product use decisions. Similarly to women, men found long-acting, discreet products that have little to no effect on sexual encounters or libido the most acceptable for their female partners’ use. Product use was most acceptable to men if they were informed of use without inadvertent discovery.

**Conclusions:**

Men’s product attribute preferences often aligned with women’s opinions of the same products. To support women’s correct use of MPTs, further research is needed to determine the best strategy for achieving male partner acceptance and support of product use, particularly for less familiar delivery forms, such as the vaginal ring.

## Introduction

Technologies for HIV prevention and family planning methods are often developed with the intention of making them female-controlled to give women greater autonomy over their sexual health [1–3]. However, navigating product use within partnerships is challenging for some women, with covert use and disclosure depending on relationship characteristics, partner communication and decision-making expectations. On the one hand, women report greater adherence when able to use products discreetly with their casual, transactional, and non-cohabitating partners [4–7], and women often appreciate products they can use discreetly or without their partner’s awareness [8–12]. On the other hand, many women prefer to share with their steady partners about the products they use [2–4, 12–15]. Male partner awareness and acceptance of microbicide product use has been shown to increase women’s own acceptance and adherence [5, 16–18], and male engagement in family planning has been shown to improve family planning acceptance, uptake, adherence, and health outcomes [19–21].

Relationship dynamics may also be affected by microbicide use and family planning. While for some couples, prevention decisions are made jointly or, are at least supported by male partners, gender norms in many settings make disclosure of HIV prevention behaviors and contraceptive use difficult. In particular, women may experience increased exposure to social harms and exacerbation of intimate partner violence (IPV) [1, 8, 22]. However, among microbicide trial participants with accepting partners, women have reported that involving their partners improved communication and increased shared responsibility for HIV protection [5, 16, 23, 24].

Little is known about male partner preferences regarding potential Multipurpose Prevention Technology (MPT) product delivery forms, that is, products that prevent unintended pregnancy as well as HIV or other sexually transmitted infections (STIs). As part of TRIO (tablet, ring and injectable as options), a study to assess end-user acceptability and preferences of three MPTs for both HIV and pregnancy prevention in Kisumu, Kenya and Soshanguve, South Africa, we explored men’s reactions to their female partner’s TRIO product use and compared men’s and women’s views of TRIO product acceptability ascertained through qualitative in-depth interviews.

## Methods

### Clinical study methods

**Fig 1:**
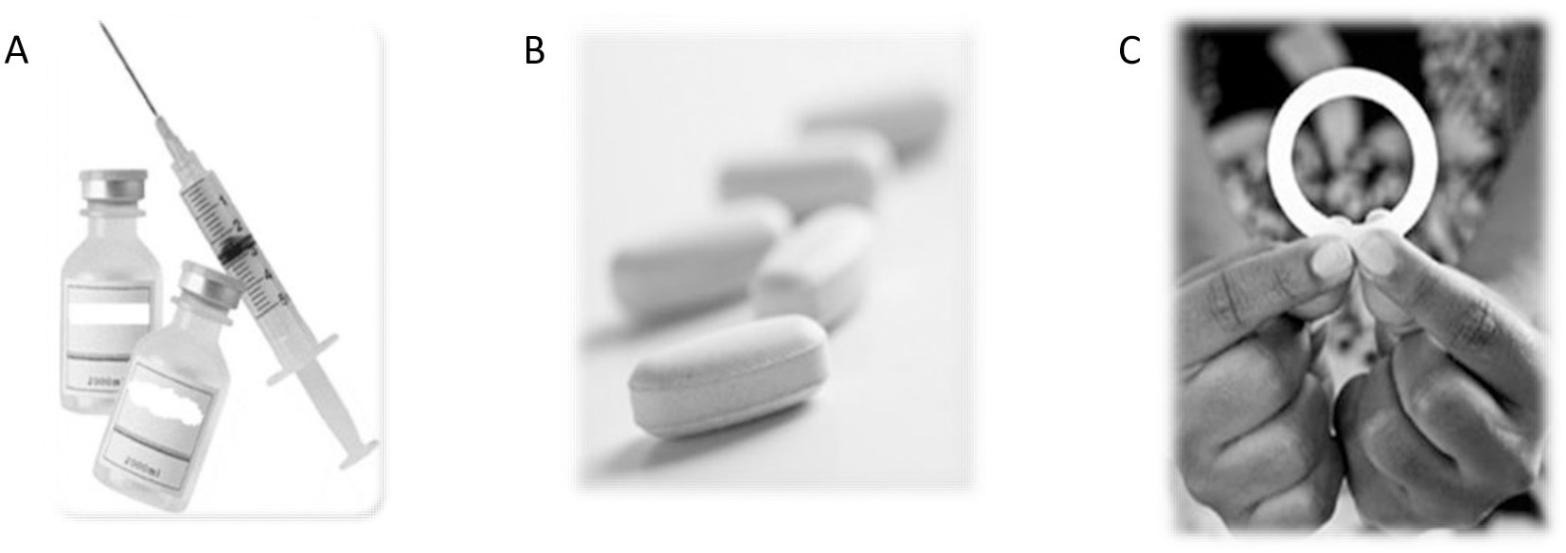
Product images. The TRIO study used three placebo delivery forms of potential Multipurpose Prevention Technology products for future prevention of pregnancy and HIV: (A) injections, (B) oral tablets, and a (C) vaginal ring.

The TRIO study took place between December 2015 and January 2017 in Kisumu, Kenya and Soshanguve, South Africa. Participants were recruited from peri-urban communities surrounding the research clinics using community mobilization and sensitization meetings, street recruitment from shopping areas, and (in South Africa only) outreach at family planning clinics and voluntary HIV counseling and testing centers. Women ages 18 to 30 were enrolled in a randomized cross-over study in which they were asked to try three placebo delivery forms of potential MPT products for future prevention of pregnancy and HIV: a vaginal ring, injections, and oral tablets; shown in Fig 1. The vaginal ring was intended to be inserted and remain in place for 4 weeks, the injectable option was once-monthly dual injections in the gluteal muscles, and the oral tablets were taken daily. After using each of these products for one month, women selected a product to use for up to two months in the second stage of the study [25].

### Qualitative methods

Qualitative data collection methods used for female TRIO clinical participants have been described in detail elsewhere [26]. During the study exit visits for TRIO clinical participants, study staff asked women for permission to contact their male partners to request an interview. Women’s disclosure status of the TRIO study regarding the purpose as well as product use was ascertained from the female participant, as well as agreement to contact their male partner, before contacting their partner to ensure conducting these interviews would not elevate the risk of social harms related to study participation. Men who consented to participate completed in-depth interviews (IDIs) of approximately 1 hour in duration in English or a local language, which were conducted by trained male interviewers using a semi-structured guide. IDIs were audio recorded, transcribed and translated into English (if needed). Interviewers were instructed to avoid asking questions that would cover topics beyond what female TRIO participants had disclosed to their male partner to ensure confidentiality was maintained. This was accomplished by asking male participants about their knowledge of the TRIO study prior to initiating other questions, then adjusting questions according to the knowledge participants demonstrated at the start of the interview.

The main topics covered in the male partner IDIs were their relationship with the female TRIO participant, knowledge of the TRIO study and products, product acceptability, male partner preferences between products, male partner influence on their TRIO female partner’s product use, and recommendations for male involvement in the future and for sex-differentiated product messaging.

The qualitative analysis team developed a codebook which was based on analytical approaches used in prior microbicide studies [12] and a conceptual model of HIV prevention product acceptability [13]. A team of three analysts coded all transcripts using Dedoose, a web-based software for qualitative and mixed-methods analysis. Code reports were generated for excerpts of transcripts where combinations of the disclosure, honesty (which covered issues around infidelity concerns related to product use), ring, injection, pill, barriers, and facilitator codes were applied. Two of the analysts who participated in the coding process wrote summary memos for each of these code reports and met to discuss emerging themes regarding male partner experiences regarding the products.

This study was conducted in accordance with the Declaration of Helsinki. All procedures and instruments were reviewed and approved by the KEMRI Scientific and Ethics Review Unit in Kenya and Pharma-Ethics Independent Research Ethics Committee in South Africa. All study participants provided written informed consent in a language that was understandable to them.

## Results

### Participant characteristics

Key baseline characteristics of the 39 male partners who consented to and participated in qualitative IDIs and the 88 women consented and enrolled in the qualitative component of TRIO are presented in Table 1. Median age for men was 29 (range: 18–42); marital status varied across sites. Nearly all men (94%) in Soshanguve were unmarried, compared with nearly all (96%) in Kisumu being married. Men at both sites indicated their TRIO partner was their primary partner (94% in Soshanguve and 100% in Kisumu). Two-thirds of male partners in Soshanguve reported more than twelve lifetime sexual partners, and one-fifth of the Kisumu male partners reported comparable numbers of partners. Nearly all men had ever tested for HIV (94% in Soshanguve; 100% in Kisumu). Across both sites, both men and women reported low levels of concern that they might get HIV in the next 12 months, with roughly one-third in Soshanguve and nearly one-half in Kisumu reporting they were “Not worried at all”.

**Table 1:** Baseline characteristics of women participating in the qualitative component of the Trio study and Trio participant male partners, Soshanguve, South Africa and Kisumu, Kenya.

**Women participating in the qualitative component of the Trio Study**		**Soshanguve (n = 45)**	**Kisumu (n = 43)**	**Overall (n = 88)**
**Age**	Mean, median	23.2, 24	23.2, 23	23.2, 23
	(min-max)	(18–30)	(18–29)	(18–30)
	18–29	44 (97.8)	43 (100)	87 (98.9)
	≥30	1 (2.2)	0 (0)	1 (1.1)
**Marital status n (%)**	Legally or traditionally married	1 (2.2)	17 (39.6)	18 (20.5)
	Not married	44 (97.8)	26 (60.5)	69 (78.4)
**How worried that might get HIV in next 12 months n (%)**	Not worried at all	14 (31.1)	21 (48.8)	35 (39.8)
	A little to somewhat worried	18 (40.0)	11 (25.6)	29 (33.0)
	Very to extremely worried	13 (28.9)	11 (25.6)	24 (27.3)
**Male Partners of women in the TRIO clinical study**		**Soshanguve (n = 16)**	**Kisumu (n = 23)**	**Overall (n = 39)**
**Age**	Mean, median (min-max)	28.4, 28 (19–41)	30.8, 31 (21–42)	29.8, 29 (18–42)
	18–29	10 (62.5)	10 (43.5)	20 (51.3)
	≥30	6 (37.5)	13 (56.5)	19 (48.7)
**Consider Trio partner to be primary partner n (%)**		14 (87.5)	23 (100.0)	37 (94.9)
**Completed secondary school n (%)**		10 (62.5)	13 (56.5)	23 (59.0)
**Religion n (%)**	Christian	10 (62.5)	22 (95.7)	32 (82.1)
	Muslim	1 (6.3)	0 (0)	1 (2.6)
	None	5 (31.3)	1 (4.3)	6 (15.4)
**Religious service attendance n (%)**	More than once a week	2 (16.7)	3 (13.6)	5 (14.7)
	Once a week	4 (33.3)	17 (77.3)	21 (61.8)
	Less than once a week	4 (33.3)	1 (4.5)	5 (14.7)
	Never	2 (16.7)	1 (4.5)	3 (8.8)
**Used any recreational drug in last 30 day n (%)**		3 (18.8)	0 (0.0)	3 (7.7)
**Lifetime sexual partners n (%)**	2–5	1 (6.3)	12 (52.1)	13 (33.3)
	6–12	3 (18.9)	6 (26.0)	9 (23.1)
	13–20	6 (37.6)	1 (4.3)	7 (18.0)
	>20	6 (37.5)	4 (17.4)	10 (25.6)
**Used condom last time had sex n (%)**		6 (37.5)	4 (19.0)	10 (27.0)
**Ever been tested for HIV n (%)**		15 (93.8)	23 (100.0)	38 (97.4)
**HIV status n (%)**	HIV-positive	1 (6.7)	0 (0.0)	1 (2.6)
	HIV-negative	13 (86.7)	22 (95.7)	35 (92.1)
	Don’t know	1 (6.7)	1 (4.3)	2 (5.3)
**How worried that might get HIV in next 12 months n (%)**	Not worried at all	5 (33.3)	11 (47.8)	16 (42.1)
	A little to somewhat worried	8 (53.3)	8(34.7)	16(42.1)
	Very to extremely worried	2 (13.3)	4 (17.3)	6 (15.8)

### Disclosure issues and implications for women’s product preference

Men most commonly described learning about TRIO from their partners within the first few weeks of women’s study enrollment; however, they encountered varying levels of disclosure. Some men understood TRIO as simply a program providing education and counseling on pregnancy (and sometimes additionally HIV) prevention while others described it as a clinical research study on pregnancy and/or HIV prevention. In many interviews it was clear through men’s explicit discussion of TRIO as education or pregnancy prevention-related that women may have downplayed or completely omitted the HIV prevention focus of the TRIO study to their partners to avoid conversations about HIV.

“*I think it’s about aahh… Mmm… teaching people how to—about family planning… And then research… She told me that they were taught on different ways of family planning [silence], only that*.”

*(Kisumu male partner, 31 years old)*

In some cases, men thought their partners were taking the tablets or using the ring to treat an STI or because they had outside sexual partners and wanted HIV protection (due to a misunderstanding among men about products being active vs. placebo). Some men were worried that some of their partner’s TRIO activities were actually NOT research-related, or that their partner was dishonest and not, in fact, participating in TRIO. They described having asked their partner for TRIO documents to make sure she was being truthful with them and not solely using the products as protection for outside sexual relationships. However, even after seeing study documentation from their partner, these men were still upset that information had been withheld from them, in cases of delayed, mid-study disclosure.

“*She must tell me, isn’t it? We agreed that we love each other. She must tell me that ‘I did something like this, and then what do you say?’ So if she is doing things in secret—secrets are not needed*.”

*(Soshanguve, male partner, 41 years old)*

In the absence of product use disclosure, men commonly discovered products when they felt the ring or saw pills for the first time. Those men assumed their partner was taking ARVs or medication for another health problem. The injection did not present this problem because injection use was not directly apparent to male partners. Notably, of the 88 female Trio qualitative participants, 57 (65%) stated after their month 3 visit that the injections were their most preferred product due the privacy it offered and because it lasted for a month; while only 16 participants stated that the tablets were most preferred, and the remaining 15 participants stated that the ring was most preferred. Women commonly stressed concerns around inadvertent product discovery and their preference for discreet products during interviews.

### Men’s versus women’s views of TRIO products

#### Attributes of TRIO products

Most men and women thought the ring was too large, too thick, and visually unappealing, and suggested it should be made smaller or thinner. Upon viewing the ring during the interview, men conjectured that its thickness and size would make insertion difficult and some thought the large size would cause the vagina to permanently stretch out. Many female partners had similar feelings when they saw the ring, one commenting “Oh my, I was scared…That [I] had to, to, to turn it into an 8 [instructions for ring use included turning the ring into a figure “8” for ease of insertion]. I had to insert it. What if it doesn’t stay well? What if it falls?” *(Soshanguve female partner*, *26 years old)*

On the other hand, men and women discussed feeling comfortable with or preferring the injection due to their familiarity as a delivery method for medications for a wide array of ailments as well as for family planning. Men thought this preference in particular was generational, with the injection most familiar and preferred among people born in the 1980s; whereas younger people may be open to the newer product forms such as tablets and rings.

“*Men of today or the couples of today were born in the 80s, leave alone our mother’s—in the 80s somebody will take what she is used to, like this one [injection]. They will talk about this thing because they were brought up when injection was there*.”

*(Kisumu male partner, 32 years old)*

Some men highlighted that women could use the injection without their male partner knowing, acknowledging that this was an advantage for women whose partners may not want them using the product. Although many men said they would agree to their partner using the injection, they were concerned about the pain of injection, which was a common concern voiced by women as well.

Several men and women were worried that if other people saw the tablets in their current form and color, the woman would be labeled HIV positive because the tablets look like antiretroviral (ARV) drugs.

A desire for a “normal” tablet was a major theme found among men and women. Men said the tablet should look like other tablets used for “everyday” drugs. Similarly, one female participant stated, “…after holding the tablet, [I felt] that tablet is too big, like for a person who can differentiate from other tablet, it can give you a negative idea…the difficulty that I have experienced was having to explain to everyone that these tablets is not an ARV [antiretroviral].” *(Soshanguve female partner*, *27 years old)* They discussed feeling comfortable with or liking tablets because of their familiarity as treatment for other ailments. However, men in Kisumu in particular voiced concern that the tablets were too large—larger than they would normally take for other, non-HIV related conditions. They questioned the need for the large size and reported this made swallowing the tablets difficult and uncomfortable for their partner—this was also in agreement with female partner descriptions of pill swallowing experiences.

“*I was just seeing this big things here [the pills], somebody taking this big things, yoh! … Yeah I think it’s too big… maybe they can make it smaller you know*.”

*(Soshanguve male partner, 39 years old)*

#### Effect on sex

Men also voiced a preference for normalcy regarding their sex life while using products, while this was a less common concern for women interviewed. Overwhelmingly, men, particularly in Kisumu, disliked the ring because they believed it caused or would cause mild to severe discomfort during sex. A few men discussed feeling the ring in specific sexual positions. Some men divulged that the ring reduced their sexual drive. Men with particularly strong feelings refused to have sex with their partner for the month of ring use or made their partner take out the ring prior to having sex.

"*Mmm I was feeling that there was something inside there—you do not feel like you are doing that normal sex. So you know—I mean you feel like you are not doing it right—that it hinders you from doing sex the right way*."

*(Kisumu male partner, 27 years old)*

"*Well imagine having sex and there’s a stick inside the vagina or something. The vagina, like it should be pure*."

*(Soshanguve male partner, 25 years old)*

Several men discussed wanting their partner to use the injection because it did not impact sex in a noticeable way. However, one male partner perceived the injection as lowering his partner’s sex drive and he disapproved of this aspect of the product (although in TRIO it was a placebo saline injection). Similarly, some men liked that the tablets did not negatively affect the sexual encounter. One male partner unexplainably thought the tablets allowed him to prolong his sexual encounters and he enjoyed the “performance change” (Soshanguve male partner, *30 years old*).

#### Side effects of delivery systems/devices and safety

Men and women had similar concerns about side effects of the TRIO products, despite knowing that in TRIO these were placebos; this was particularly the case for the ring and tablets. Several men stated the ring had fewer side effects or none compared to the other TRIO products. Nevertheless, men and women had many concerns related to the ring being worn vaginally for an extended period of time. Many men simply could not conceive of a product that was vaginally inserted, let alone continually worn for a month. While most men were able to articulate a specific fear related to vaginal insertion or continual use of the ring, several just described a general dislike for a vaginal product.

"*I don’t like it totally… Because of where it is being put, I don’t like it—from the word go… I don’t like this ring*."

*(Kisumu male partner, 32 years old)*

Some women similarly felt disconcerted by use of the ring. One woman stated,

“*It’s just that, it was always on my mind that I have the ring on and the way it was on my mind I was not comfortable…So I felt that even if I squat and I am not comfortable*.”

*(Kisumu female partner, 30 years old)*

Men and women shared major concerns that the ring could be pushed too far up during sex, especially rough sex, fearing the ring could get lost, stuck, or disappear. One man shared, *"That is what was disturbing me*, *that it could go too deep*.*" (Kisumu male partner*, *41 years old)* Additionally, men and women were concerned with the ring not being able to stay in place and falling out, especially during strenuous activities. Several men described the ring coming out during sex. However, many assumed this meant their partner had not inserted the ring correctly.

Regarding tablets, Kisumu men shared unique concerns about side effects that had not been shared by women participants. The two greatest concerns raised by these men were potential stomach problems and general harm from too much medicine in the body. Some men felt that taking the tablets daily would be “too much” for the body, lead to drug accumulation or to other medications not working because of drug resistance. *"When you take a lot of medicine you may develop resistance so that when you take other medicine like anti-malarial*, *you don’t get well*.*" (Kisumu male partner*, *34 years old)*

#### Dosing and adherence

Many men were generally supportive of their partner using TRIO products because they wanted their partner to one day have protection from pregnancies and/or HIV. Due to this support, they showed great concern about ensuring their partner found a product that they could adhere to, and in some cases, expressed how they prefer to be personally involved to monitor -or control—their partner’s adherence to a product. So in spite of men’s negativity about the ring’s perceived effect on their sexual life, some men spoke positively about their partner’s likelihood of achieving higher adherence with the ring, especially in comparison to the pill. *"I like it the most*. *The vaginal ring*, *it’s a once off*, *it’s something you won’t forget*.*" (Soshanguve male partner*, *27 years old)*

Similarly, men and women liked the longer-lasting protection and associated ease of use that the injection would offer. Sharing an opinion common among women interviewed, one woman commented that, *“In my own thoughts*, *I can say that the injection is good for using…It is easy to use*.” *(Kisumu female partner*, *25 years old)*. Additionally, six men independently suggested the injection should offer longer-acting protection ranging from three to six months because going to the clinic every month (the dosing regimen in TRIO) and the fear of pain with the injection were perceived barriers to adherence. These opinions about barriers to use were shared by women participants as well.

Overwhelmingly, men and women were concerned about the feasibility of adhering to a daily oral dosing regimen, and described difficulties with the daily burden of taking a tablet. Several brought up specific concerns that missing doses could leave women at risk of contracting HIV, becoming pregnant, or developing “drug resistance”. Several men noted that they could help by reminding their partner to take the pills, which women saw as a positive form of support; however, men still had lingering concerns about the repercussions of non-adherence.

“*I think the negative is that, using a pill can also increase your chance of falling pregnant should she not take the pill on time, so that’s a negative. Because now, what if both of us forget ‘cause I have to remind her and what if she forgets, she doesn’t remember to take the pill and I also don’t remind her to take the pill, then she can fall, she can fall pregnant. The pill, it’s a bit of high risk for, for a couple like, for like us because we, we [are] not good at taking medication*.”

*(Soshanguve male partner, 30 years old)*

Men thought it was inevitable that a person will struggle with adherence to daily tablets and suggested researchers develop pills that offer protection for a week or a month to address this challenge.

## Discussion

In this qualitative study, we explored men’s reactions to their female partner’s TRIO product use -Tablets, Ring and Injections- and compared men’s and women’s views of TRIO product acceptability. Men’s opinions were often similar to women’s opinions of products—men expressed a desire to know of their partner’s product use decisions and about the effects of products on sexual encounters with their partner [26]. However, somewhat unexpectedly, men expressed a high level of concern regarding maximizing the ease of product adherence for their partner.

The discreetness of a product had tangible effects on how men learned of product use and their early impressions of products. Products that were prone to inadvertent discovery—the tablets and the vaginal ring—left open the opportunity for men to find out about them and draw their own conclusions about the product before women had the chance to provide an explanation about them. Men drew various conclusions on their own as a result, such as thinking women were taking the tablets or using the ring for a secret motive, such as to treat an STI or HIV, or to use the products as protection while cheating on them. Men and women discussed both hypothetical and real instances of these situations causing tension in their relationships and anxiety surrounding this, similar to other research findings [7]. Both men and women spoke positively about discreet products—such as the injections—with which male partner discovery and suspicion was likely to be averted. This aligns with previous research findings regarding women’s preferences for discretion [8–12].

Men’s narratives around interaction with product use indicated a desire for wanting to be aware of and involved with product use decisions at the outset. However, this desire for involvement sometimes revealed a desire to control their female partner’s product use activities. These findings are supported by other qualitative work indicating that that women would “face problems at home” if they did not first ask permission to use a product [16] and that men often prefer to be the “decision-makers” in matters of sex and reproductive health [27, 28].

In terms of product attribute preferences, men primarily desired ‘normalcy’ in a product, i.e., a product with no effect on sexual encounters or pleasure, not being confronted with something outside their realm of previous experience, and that also facilitated their partner’s adherence. Men’s concerns regarding a product’s effect on sex is corroborated by other work which has found that women were concerned with microbicides negatively impacting the partner’s sexual pleasure and partner approval [3, 15, 29]. Men took greatest issue with products that they saw as having an impact on their sexual experiences, with the vaginal ring being the most cited product in this regard. However, this negative opinion on the ring was tempered by their recognition that the ring would require less effort for their partners to sustain high adherence, which they viewed positively. Across products, men contrasted the longer acting (ring and injection) and shorter acting product (tablet) in a context of ease of use and the differing levels of effort needed on their part to support adherence, thereby viewing both the ring and injection positively due to their long-acting nature.

### Limitations

Several design limitations should be considered when evaluating the findings reported here. First, as noted previously, there were significant differences by site in terms of the product preferences and choices from the female partners’ perspective; however, we were not able to compare men and women’s opinions quantitatively because only qualitative information on preferences was collected among men [30]. Additionally, the men interviewed for this component of the study were a convenience sample determined by their female partner’s willingness for their partner to be contacted, so this is likely a more supportive and thus biased sample. Nonetheless, some partners interviewed knew very little about the TRIO Study indicating that the female TRIO participants had opted not to disclose product use to them, nor let this deter them from volunteering their partner’s contact information for the IDIs. Though the products used by women were placebos and, hence, side effects of active drugs were not evaluable as part of the preference assessment, this design feature allowed the assessments of perceptions and preferences to focus on the product delivery form without side effects of the active drugs being part of the evaluation of products.

## Conclusions

In the TRIO study, men found long-acting, discreet products that have little to no effect on sexual encounters or libido the most acceptable for their female partners’ use. Furthermore, men found product use more acceptable if they were informed of its’ use prior to discovery (without disclosure). To support women’s use of MPTs and increase adherence, further research is needed to determine the best method for achieving male partner acceptance and support of product use, particularly for less familiar delivery forms, such as the vaginal ring.

## Supporting information

S1 File Trio study clinical study Demographic form (DEM). Demographic questionnaire for female participants.
(PDF)

S2 File Trio study Male Partners Demographic form (MP DEM). Demographic questionnaire for male partner participants.
(PDF)

S3 File Trio study In-depth Interview (IDI) guide for female clinical study participants, round 1. IDI guide for first round female participant interviews.
(PDF)

S4 File Trio study In-depth Interview (IDI) topic guide for female clinical study participants, round 2 (Exit). IDI guide for second round female participant interviews.
(PDF)

S5 File Trio male partner In-depth Interview (IDI) topic guide. IDI guide for male partner interviews.
(PDF)
