# Supplementary material for: Female and male partner perspectives on placebo Multipurpose Prevention Technologies (MPTs) used by women in the TRIO study in South Africa and Kenya
Source: PLoS One. 2022 May 12;17(5):e0265303. doi: 10.1371/journal.pone.0265303 (PMC9097999; doi:10.1371/journal.pone.0265303)
Supplement: S1 File — Demographic questionnaire for female participants. (PDF) [file pone.0265303.s001.pdf]

___ ___ ___ ___
Participant ID

______.___
Visit Code

__ __ / __ __ / __ __ __ __
Today's date (dd/mm/yyyy)

***Instructions**: Complete at enrolment visit. Unless otherwise indicated, only one response may be selected. Italicized text should not be read to participants.*

Demographic		
1.	What is your date of birth?	☐☐/☐☐/☐☐ *or*<br>*day month year*<br>*If unknown, record age*: ☐☐
2.	What is your race or ethnic group? (*mark race/ethnic group code*)	☐☐ *Race/Ethnic Group Code*<br>☐ *Other, specify*: ________________
3.	What is the language most spoken at home? (*mark language code*)	☐☐ *Language code*<br>☐ *Other, specify*: ____________
4.	Name of area/location where you currently live:	__________________________
5.	For how long have you lived in this location/area? (*mark one*)	☐☐ *or* ☐☐<br>*months* *years*
6.	Do you consider this your home?	☐1 *Yes*<br>☐2 *No, specify where home is:*<br>_________________________
7.	Are you currently married? *(Read responses.)*	☐1 Yes, legally married<br>☐2 Yes, traditionally married<br>☐3 No<br>☐4 Other, *specify*:___________

8. Who are the people you live with now?	*Yes*	*No*
8a. I live alone	☐	☐
	If lives alone, skip to item 9	
8b. Mother and/or father	☐	☐
8c. Sibling(s)	☐	☐
8d. Grandparent(s)	☐	☐
8e. Other relative(s)	☐	☐
8f. Your child(ren)	☐	☐
8g. Friend(s)/Roommate(s)	☐	☐
8h. Spouse or boyfriend	☐	☐
8i. Other, *specify*: ________________	☐	☐

Staff initials:___________

___ ___ ___ ___ Participant ID ______.___ Visit Code __ __ / __ __ / __ __ __ __ Today's date (dd/mm/yyyy)

9.	How many total children are you currently taking care of? Include your own children as well as other children that you are responsible for, not including children cared for at work.	☐☐	
10.	Do you, or does someone in your family, own the household you are currently living in?	☐1 *Yes*<br>☐2 *No*	
11.	Is there a place in your home that you can go where you know you will have privacy?	☐1 *Yes*<br>☐2 *No*	
12.	Dose your household have…?	*Yes*	*No*
	12a. Electricity	☐	☐
	12b. A radio	☐	☐
	12c. A television	☐	☐
	12d. A mobile telephone	☐	☐
	12e. A non-mobile telephone	☐	☐
	12f. A refrigerator	☐	☐
13.	What kind of toilet facility does your household have? *(mark toilet facility code)*	☐☐ *Toilet Facility Code*<br>Other, *specify*:__________________	
14.	What is the main source of drinking water for members of your household? *(mark water source code)*	☐☐ *Water Source Code*<br>Other, *specify*:__________________	
15.	What is your highest level of education? *(Read responses.)*	☐1 No schooling<br>☐2 Primary school, not complete<br>☐3 Primary school, complete<br>☐4 Secondary school, not complete<br>☐5 Secondary school, complete<br>☐6 Attended college or university	
16.	Do you currently receive your own source of income?	☐1 *Yes*<br>☐2 *No* → ***skip to item 18***	
17.	What is the source of your current income?	*Yes*	*No*
	17a. Formal employment	☐	☐
	17b. Self-employment	☐	☐
	17c. Government grant(s)	☐	☐
	17d. Other, *specify*: ____________________	☐	☐
18.	In the past four weeks, how often did you worry that you would not have enough food? *(Read responses.)*	☐1 Never<br>☐2 Rarely (once or twice)<br>☐3 Sometimes (3-10 times)<br>☐4 Often (more than 10 times)	

Staff initials:_____________

___ ___ ___ ___ Participant ID　　______.___ Visit Code　　__ __ / __ __ / __ __ __ __ Today's date (dd/mm/yyyy)

19.	In the past four weeks, how often did you have no food to eat of any kind in your household? *(Read responses.)*	☐1 Never<br>☐2 Rarely or sometimes<br>☐3 Often
20.	In the past four weeks, how often did you go to sleep at night hungry? *(Read responses.)*	☐1 Never<br>☐2 Rarely or sometimes<br>☐3 Often
21.	In the past four weeks, how often did you go a whole day and night without eating? *(Read responses.)*	☐1 Never<br>☐2 Rarely or sometimes<br>☐3 Often
22.	What is your religion? *(Read responses.)*	☐1 Christian<br>☐2 Muslim<br>☐3 Other, *specify*: ______________<br>☐4 None → ***skip to item 24***
23.	How many times a week do you attend religious services? *(Read responses.)*	☐1 More than once a week<br>☐2 Once a week<br>☐3 Less than once a week<br>☐4 Never

Substance Use		
Interviewer Reads: Now I will ask you about your alcohol and drug use behaviors.		
24.	During the last 30 days, how often did you have any kind of drink containing alcohol? By a drink, we mean a 330mL can or glass of beer or cooler, a glass of wine, or a drink containing 1 shot of liquor.	☐1 *Every day*<br>☐2 *5-6 times a week*<br>☐3 *3-4 times a week*<br>☐4 *Twice a week*<br>☐5 *Once a week*<br>☐6 *2-3 times a month*<br>☐7 *Once a month*<br>☐8 *I did not drink alcohol in the past 30 days, but have drunk alcohol in my life*→ ***skip to item 27***<br>☐9 *I never drank alcohol in my life*→ ***skip to item 27***
25.	During the last 30 days, how many alcoholic drinks did you have on a typical day when you drank alcohol?	☐☐ *drinks*
26.	During the last 30 days, how often did you have 4 or more drinks containing any kind of alcohol within a two-hour period?	☐1 *Every day*<br>☐2 *5-6 times a week*<br>☐3 *3-4 times a week*<br>☐4 *Twice a week*<br>☐5 *Once a week*<br>☐6 *2-3 times a month*<br>☐7 *Once a month*<br>☐8 *I did not do this in the past 30 days, but have in the past*<br>☐9 *I've never done this in my life*

Staff initials:_____________

___ ___ ___ ___ Participant ID   ______.___ Visit Code   __ __ / __ __ / __ __ __ __ Today's date (dd/mm/yyyy)

27.	In the last 30 days, have you smoked, swallowed, snorted, or injected any kind of recreational drug? Do not include tobacco or cigarettes.	☐1 *Yes, specify drug*: ______________________<br>☐2 *No*

Sexual History		
Interviewer Reads: The next questions ask about your sexual behavior and sexual partners.		
28.	How many sexual partners have you had in your lifetime? By sexual partner we mean someone with whom you have had vaginal, anal or oral sex.	☐☐☐ *Specify number*
29.	In your lifetime, have you and your partner(s) ever used condoms for vaginal sex?	☐1 *Yes*<br>☐2 *No* → ***skip to item 32***
30.	In the past 30 days, was a condom ever used when you had vaginal sex?	☐1 *Yes*<br>☐2 *No*<br>☐3 *N/A, no sex in past 30 days*
31.	The last time you had vaginal sex, was a condom used?	☐1 *Yes*<br>☐2 *No*
32.	In your lifetime, have you ever received money, goods, a place to stay, or services in exchange for sex?	☐1 *Yes*<br>☐2 *No* → ***skip to item 34***
33.	In the past 30 days, have you received money, goods, a place to stay, or services in exchange for sex?	☐1 *Yes*<br>☐2 *No*
34.	In total, how many sexual partners have you had in the past 30 days?	☐☐☐ *Specify number*
35.	Do you currently have a primary partner? By primary partner, I mean a husband, boyfriend, or steady partner with whom you regularly have sex.	☐1 *Yes*<br>☐2 *No* → ***skip to item 39***
36.	Are you living with this partner?	☐1 *Yes*<br>☐2 *No*
37.	For how long have you been together with this partner? (*mark one*)	☐☐ *Specify months or*<br>☐☐ *Specify years*
38.	Do you believe your primary partner has sexual partners other than you? *(Read responses.)*	☐1 Yes, I know<br>☐2 Yes, I suspect<br>☐3 No<br>☐4 Don't know
39.	Do you currently have casual sex partner(s)? A casual partner is someone with whom you have sex but not a committed relationship.	☐1 *Yes*<br>☐2 *No*

___ ___ ___ ___ Participant ID

______.___ Visit Code

__ __ / __ __ / __ __ __ __ Today's date (dd/mm/yyyy)

40.	i. What methods for family planning and/or HIV prevention have you **ever** used? ii. *For each method ever used, ask* "Have you used this in the **past 30 days**?"	i. Ever		ii. Past 30 days	
		Yes	*No*	*Yes*	*No*
	40a. Male condom	☐1	☐2	☐1	☐2
	40b. Female condom	☐1	☐2	☐1	☐2
	40c. Diaphragm	☐1	☐2	☐1	☐2
	40d. Gel	☐1	☐2	☐1	☐2
	40e. Pills	☐1	☐2	☐1	☐2
	40f. IUD	☐1	☐2	☐1	☐2
	40g. Implants	☐1	☐2	☐1	☐2
	40h. Sterilization (female)	☐1	☐2	☐1	☐2
	40i. Sterilization (male)	☐1	☐2	☐1	☐2
	40j. Injectable	☐1	☐2	☐1	☐2
	40k. Traditional/rhythm method	☐1	☐2	☐1	☐2
	40l. Other (*specify*): ______________________	☐1	☐2	☐1	☐2

Vaginal Practices

Interviewer Reads: We know that many women insert different products in the vagina for a variety of purposes. We want to know about your experiences with using different products in your vagina.

41.	In the last 3 months, have you used any of the following to control or manage menstrual blood or spotting?	*Yes*	*No*
	41a. Tissue, toilet paper, cloth or cotton wool put inside the vagina	☐1	☐2
	41b. Tissue, toilet paper, cloth or cotton wool paced in underwear/clothing	☐1	☐2
	41c. Tampon	☐1	☐2
	41d. Sanitary pad	☐1	☐2
	41e. Water without soap, inside the vagina	☐1	☐2
	41f. Water with soap, inside the vagina	☐1	☐2
	41g. Anything else? *Specify*:________________________	☐1	☐2

Staff initials:_____________

___ ___ ___ ___ Participant ID

_______.____ Visit Code

__ __ / __ __ / __ __ __ __ Today's date (dd/mm/yyyy)

		Yes	*No*
42.	In the past 3 months, have you put any of the following inside your vagina at times other than during your menses?		
	42a. Water only	☐1	☐2
	42b. Water plus soap	☐1	☐2
	42c. Fingers, to clean or insert something	☐1	☐2
	42d. Materials such as paper, cloth, or cotton wool	☐1	☐2
	42e. Other materials or substances to tighten or dry the vagina	☐1	☐2

Risk Perception		
Interviewer Reads: Now I'm going to ask you questions about how worried you are about getting HIV or having an unplanned pregnancy. I'll ask you a question and read several possible answers for you to choose from. Choose only one answer for each question.		
43.	How worried are you that you might get HIV in the next 12 months? *(Read responses.)*	☐1 Not worried at all<br>☐2 A little worried<br>☐3 Somewhat worried<br>☐4 Very worried<br>☐5 Extremely worried
44.	In the past 12 months, is getting HIV something you have…? *(Read responses.)*	☐1 Never thought about<br>☐2 Rarely thought about<br>☐3 Thought about some of the time<br>☐4 Thought about often
45.	How likely is it that you will become infected with HIV in the next 12 months? *(Read responses.)*	☐1 Extremely unlikely<br>☐2 Very unlikely<br>☐3 Somewhat likely<br>☐4 Very likely<br>☐5 Extremely likely
46.	How likely is it that you will have an unplanned pregnancy in the next 12 months? *(Read responses.)*	☐1 Extremely unlikely<br>☐2 Very unlikely<br>☐3 Somewhat likely<br>☐4 Very likely<br>☐5 Extremely likely
47.	If you were to not use any contraceptive method, how likely is it that you would become pregnant in the next 12 months? *(Read responses.)*	☐1 Extremely unlikely<br>☐2 Very unlikely<br>☐3 Somewhat likely<br>☐4 Very likely<br>☐5 Extremely likely

Staff initials:_____________
