# Supplementary material for: Female and male partner perspectives on placebo Multipurpose Prevention Technologies (MPTs) used by women in the TRIO study in South Africa and Kenya
Source: PLoS One. 2022 May 12;17(5):e0265303. doi: 10.1371/journal.pone.0265303 (PMC9097999; doi:10.1371/journal.pone.0265303)
Supplement: S2 File — Demographic questionnaire for male partner participants. (PDF) [file pone.0265303.s002.pdf]

___ ___ ___ ___
Participant ID

___ ___ / ___ ___ / ___ ___
Today's date (dd/mm/yy)

Instructions*: Complete at enrolment prior to conducting the in-depth interview..*

**Demographic**		
1.	What is your date of birth?	☐☐/☐☐/☐☐ *or*<br>day month year<br>If unknown, record age: ☐☐
2.	What is your race or ethnic group? (*mark race/ethnic group code*)	☐☐ Race/Ethnic Group Code<br>Other, specify:<br>__________________
3.	What is the language most spoken at home? (*mark language code*)	☐☐ Language Code<br>☐ Other, specify:<br>__________________
4.	Name of area/location where you currently live:	____________________________
5.	For how long have you lived in this location/area? (*mark one*)	☐☐ or ☐☐<br>months years
6.	Are you currently living with your partner who participated in the Trio study?	☐ Yes<br>☐ No
7.	Are you currently married?	☐ Yes, legally married<br>☐ Yes, traditionally married<br>☐ No<br>☐ Other, specify:_______________

8.	Who are the people you live with now?<br>[*Probe: Anyone else?]*		Yes	No
		Mark all that apply.		
		Mother and/or father	☐	☐
		Sibling(s)	☐	☐
		Grandparent(s)	☐	☐
		Other relative(s)	☐	☐
		Your child(ren)	☐	☐
		Friend(s)/Roommate(s)	☐	☐
		Spouse or girlfriend	☐	☐
		If no to all in list above, mark yes to alone OR mark yes to "other" and specify below:		
		Alone	☐	☐
		Other, specify:<br>__________________	☐	☐

___ ___ ___ ___ ___ ___ / ___ ___ / ___ ___
Participant ID Today's date (dd/mm/yy)

9.	How many total children are you currently taking care of? Include your own children as well as other children that you are responsible for, not including children cared for at work.	☐☐		
10.	Do you, or does someone in your family, own the household you are currently living in?	☐ Yes<br>☐ No		
11.	Does your household have:…?		Yes	No
		Electricity	☐	☐
		A radio	☐	☐
		A television	☐	☐
		A mobile telephone	☐	☐
		A non-mobile telephone	☐	☐
		A refrigerator	☐	☐
12.	What kind of toilet facility does your household have? *(mark toilet facility code)*	☐☐ Toilet Facility Code<br>Other, specify:___________________		
13.	What is the main source of drinking water for members of your household? *(mark water source code)*	☐☐ Water Source Code<br>Other, specify:___________________		
14.	How many years of school have you completed?	☐☐ years		
15.	Do you currently earn an income of your own?	☐ Yes<br>☐ No → **skip to item 17**		
16.	How do you earn your current income?		Yes	No
		Formal employment	☐	☐
		Self-employment	☐	☐
		☐ Other, specify: _______________		
17.	What is your religion?	☐ Christian<br>☐ Muslim<br>☐ Other, specify: _______________<br>☐ None → **skip to item 19**		
18.	How many times a week do you attend religious services?	☐ More than once a week<br>☐ Once a week<br>☐ Less than once a week<br>☐ Never		

Staff initials:_____________

___ ___ ___ ___
Participant ID

___ ___ / ___ ___ / ___ ___
Today's date (dd/mm/yy)

**Substance Use**		
**Interviewer Reads:** Now I will ask you about your alcohol use behaviors.		
19.	During the last 30 days, how often did you usually have any kind of drink containing alcohol? By a drink we mean half an ounce of absolute alcohol (e.g., a 12 ounce can or glass of beer or cooler, a 5 ounce glass of wine, or a drink containing 1 shot of liquor).	☐ Every day<br>☐ 5-6 times a week<br>☐ 3-4 times a week<br>☐ Twice a week<br>☐ Once a week<br>☐ 2-3 times a month<br>☐ Once a month<br>☐ I did not drink alcohol in the past 30 days, but have drunk alcohol in my life<br>☐ I never drank alcohol in my life→ **skip to item 22**
20.	During the last 30 days, how many alcoholic drinks did you have on a typical day when you drank alcohol?	☐☐ drinks
21.	During the last 30 days, how often did you have 4 or more drinks containing any kind of alcohol in within a two-hour period?	☐ Every day<br>☐ 5-6 times a week<br>☐ 3-4 times a week<br>☐ Twice a week<br>☐ Once a week<br>☐ 2-3 times a month<br>☐ Once a month<br>☐ I did not do this in the past 30 days, but have in the past<br>☐ I've never done this in my life
22.	In the last 30 days, have you smoked, swallowed, snorted, or injected any kind of recreational drug? Do not include tobacco or cigarettes.	☐ Yes (specify):________________<br>☐ No
**Sexual History**		
**Interviewer Reads:** The next questions ask about your sexual behavior and partners.		
23.	How many sexual partners have you had in your lifetime? By sexual partner we mean someone with whom you have had vaginal, anal or oral sex.	☐☐☐ Specify number
24.	In your lifetime, have you and your partner(s) ever used condoms for vaginal sex?	☐ Yes<br>☐ No → **skip to item 27**
25.	In the past 30 days, was a condom ever used when you had vaginal sex?	☐ Yes<br>☐ No<br>☐ N/A, no sex in past 30 days
26.	The last time you had vaginal sex, was a condom used?	☐ Yes<br>☐ No

Staff initials:_____________

___ ___ ___ ___
Participant ID

___ ___ / ___ ___ / ___ ___
Today's date (dd/mm/yy)

27.	For how long have you been together with your partner who participated in the Trio study?<br>(*mark one*)	☐☐ Specify months<br>*or*<br>☐☐ Specify years
28.	Do you consider her to be your primary partner? By primary partner, I mean a wife, girlfriend, or steady partner with whom you regularly have sex.	☐ Yes<br>☐ No
29.	In the past 30 days, how many times did you have vaginal sex with your partner who participated in the Trio study?	☐☐ Specify number
30.	In the past 30 days, how many other sex partners did you have besides your partner who participated in the Trio study?	☐☐ Specify number
31.	Which of the following describes how often you have sex in a month?	☐ Daily or almost every day<br>☐ About once a week<br>☐ Once or twice in a month<br>☐ I don't have a normal pattern<br>☐ Other, specify_______________
32.	Are you circumcised?	☐ Yes<br>☐ No<br>☐ Not sure
33.	Have you ever been tested for HIV?	☐ Yes<br>☐ No → **skip to item 34**
34.	What is your HIV status?	☐ HIV-positive → **END FORM**<br>☐ HIV-negative<br>☐ Don't know<br>☐ Refused to answer
**Risk Perception**		
**Interviewer Reads:** Now I'm going to ask you questions about how worried you are about getting HIV. I'll ask you a question and read several possible answers for you to choose from. Choose only one answer for each question.		
35.	How worried are you that you might get HIV in the next 12 months?	☐ Not worried at all<br>☐ A little worried<br>☐ Somewhat worried<br>☐ Very worried<br>☐ Extremely worried
36.	In the past 12 months, is getting HIV something you have [*read options*]?	☐ Never thought about<br>☐ Rarely thought about<br>☐ Thought about some of the time<br>☐ Thought about often
37.	How likely is it that you will become infected with HIV in the next 12 months?	☐ Extremely unlikely<br>☐ Very unlikely<br>☐ Somewhat likely<br>☐ Very likely<br>☐ Extremely likely

Staff initials:_____________
