# Supplementary material for: Female and male partner perspectives on placebo Multipurpose Prevention Technologies (MPTs) used by women in the TRIO study in South Africa and Kenya
Source: PLoS One. 2022 May 12;17(5):e0265303. doi: 10.1371/journal.pone.0265303 (PMC9097999; doi:10.1371/journal.pone.0265303)
Supplement: S3 File — IDI guide for first round female participant interviews. (PDF) [file pone.0265303.s003.pdf]

Trio Study

In-depth Interview (IDI) Guide for Female Clinical Study Participants, Round 1

INSTRUCTIONS for the Interviewer: How to use the IDI Guide

1. There are two levels of questions:

   • Primary interview questions: appear in **bold** text. They address the topics that you as the interviewer must ask and discuss with participants. The questions are suggestions for getting the discussion going. You are not required to read them verbatim, but they are written to ensure some consistency across IDIs. You may adapt the questions and/or ask them in a different order, depending on how the interview develops. However you will have to ensure that by the end of the interview, all the topics and key themes have been covered.

   • Probing topics: are indicated with a bullet. If you find that the participant provides little information in response to the primary question, these probing topics may be used to encourage further discussion. You are not required to cover every topic listed. So, depending on what has already been discussed, and the IDI context, you may ask these probes or not.

2. *Instructions/suggestions to interviewer are in italics and [brackets].*

3. Words found in (parentheses) are meant to provide wording options to interviewers to fit various situations. For example, they often provide a present or past tense verb.

4. The IDI guide is not meant to be used to take notes. Rather, you should use the separate notes form, where you will also insert your initials, the participant's PTID, as well as the date, start and end time of the interview.

[*Start Recorder and Read Introduction*]: My name is _________________. Thank you again for your willingness to be a part of this discussion. I am looking forward to hearing your thoughts on the products you have been using. If during our discussion, there are issues or concerns that you would like to talk about, feel free to bring them up, even if I didn't ask about them. If you have specific questions during the interview, I will take note of them and answer them directly after the interview. If I cannot answer them, I can refer you to someone who may be able to help.

The main goal of this discussion is to better understand your experience with the products you have used so far in the Trio study. What you share will help scientists create products that women like you will actually want to use, so we especially want to hear if there was anything you didn't like about the products, so that we can improve them.

Warm-up and Study Experience
**First we are going to talk about your experience being part of this study so far…**
1. **Tell me about your experience being part of Trio so far (*Do not discuss specific products with her).*** *Possible probing topics:* • Experiences with workshop – how did the workshop make you feel about being part of the study? • What (other) activity(s) helped and what activities didn't help you feel connected, engaged, and excited about the study? (SMS, video, educational materials, product counseling) • What suggestions do you have for the researchers to make participants feel more engaged? • Challenges to participating (individual, relationship/social, community level challenges) • Experiences during study visits or at the clinic, including interactions with other participants • Effects of study participation on daily life for participant, partner(s) and others • Changes in relationship with male partner(s), including decision-making ability and communication around pregnancy and HIV prevention

Product Adherence and Acceptability
**Now we are going to talk more about your experiences using <u>the tablets</u> during this study…**
2. **How would you describe your reaction when you saw the tablets for the first time and over the month you used them?** *Possible probing topics:* • How did feelings about the tablets change over time? • Any changes after touching or holding the tablets? • Any changes after first swallowing a tablet? • Any changes after having used the tablets for a while?
3. **What did you like and dislike about using the tablets?** *Possible probing topics:* • Negative / undesirable features (taste, smell, size, color, texture) • Medication and/or HIV stigma • Positive / desirable features (taste, smell, size, color, texture) • Convenience/ ease of use and of storage • What do you like and dislike most about the tablets? • Is there anything you would change about the tablets that would make you more likely to use them in the future? • Would you prefer a tablet you take around the time of sex rather than daily? • Would you prefer taking HIV prevention and family planning tablets separately?

4. **On the days that you didn't take the tablet, what were your reasons for not taking it?**

Possible probing topics:

- How did you think you were supposed to use the tablets
- Difficulty taking the tablets every day
- What helped you to take the tablets (food, EM system, SMS, other reminders, social support)?

5. **Who did you talk to about taking the tablets?**

Possible probing topics:

- Who knew you were taking the tablets? What was their reaction?
- How and with whom did you make decisions around tablet use (e.g. male partner, family members, other participants)
- Who didn't you tell about taking tablets? Why didn't you tell them? How did you feel about the possibility of them finding out about it?

Now we are going to talk more about your experiences with the injections during this study…

6. **How would you describe your reaction when you saw the injections for the first time and over the month after you received them?**

Possible probing topics:

- How did feelings about the injections change over time?
- Any changes just after receiving the injections?
- Any changes after a month of having received the injections?

7. **What do you like and dislike about receiving the injections?**

Possible probing topics:

- Negative / undesirable features (needle size, feeling, location - frequency and number of shots; stigma; need to go to clinic to get injection)
- Positive / desirable features (clinician administered, needle size, feeling, location & frequency of shots; perceived efficacy; regimen: only once every 2-3 months; rather discreet method)
- Convenience/ ease of use
- What do you like and dislike most about the injections?
- Is there anything you would change about the injections that would make you more likely to use them in the future?

8. **What was it like to get the injections once in a month?**

Possible probing topics:

- How did the injections fit in with your routine or lifestyle?

9. **Who did you talk to about getting the injections?**

Possible probing topics:

- Who knew you were using the injections? What was their reaction?
- Who didn't you tell about using the injections? Why didn't you tell them? How did you feel about the possibility of them finding out about it?

Now we are going to talk more about your experiences using the ring during this study…

10. **How would you describe your reaction when you saw the ring for the first time?**

Possible probing topics:

- How did feelings about the ring change over time?
- Any changes after touching or holding the ring?
- Any changes after first inserting the ring?
- Any changes after having used the ring for a while?

11. **What did you like about the ring? What did you dislike? Why?**

Possible probing topics:

- Negative / undesirable features (size, feeling, shape, color, vaginal insertion, hygiene, stigma, interference with sex)
- *IF feeling mentioned:* feeling upon initial insertion and once inside; situations when you were more or less aware of the ring
- Positive / desirable features (who inserts it, feeling, duration of use, perceived efficacy, discreet method)
- Convenience/ease of use; insertion and removals (or lack thereof)
- What do you like and dislike most about the ring?
- Is there anything you would change about the ring that would make you like it more?

12. **How easy or difficult was it to wear the ring for a month?**

Possible probing topics:

- How did wearing the ring fit in with your daily routine or lifestyle?
- Ease/difficulty of wearing the ring continuously between visits
- Ease/difficulty of inserting and removing the ring
- Use during menses
- Tell me about all the times when you took the ring out – what was going on (description of circumstances, outcome)
- Tell me about all the times it came out on its own – what was happening

13. **How did using the ring affect sex?**

Possible probing topics:

- Ring removals before or during sex
- Awareness of the ring during sex for you and partner
- Positive or negative physical or emotional changes with sex
- Changes in sexual practices, sexual partners, frequency, or reasons for having sex, etc.

14. **Who did you talk to about wearing the ring?**

Possible probing topics:

- Who knew you were using the ring? What was their reaction?
- Partner's reaction and attitude
- How and with whom did she make decisions around ring use (e.g. male partner, family members, other participants)
- Who didn't you tell about using the ring? Why didn't you tell them? How did you feel about the possibility of them finding out about it?

Product Preferences

15. **Think about other methods you have used to prevent pregnancy or HIV in the past. What did you like and dislike about these methods?**

Possible probing topics:

- Why did you first try these products?
- Where did you learn about the products you tried?
- Ever used condoms?
- How much did they interfere with sex, life, or regular activities?
- If there are methods you tried, but then stopped using, why did you stop using these methods?

16. **How does the tablet compare with other products you have used to prevent pregnancy or HIV?**

Possible probing topics:

- How do tablets compare to condoms? What makes them easier or harder to use?
- How much does it interfere with sex, life, or regular activities compared to other methods that you have tried?

17. **How do the injections compare with other products you have used to prevent pregnancy or HIV?**

Possible probing topics:

- How do the injections compare to condoms? What makes them easier or harder to use?
- How much does it interfere with sex, life, or regular activities compared to other methods that you have tried?

18. **How does the ring compare with other products you have used to prevent pregnancy or HIV?**

Possible probing topics:

- How does the ring compare to condoms? What makes it easier or harder to use?
- How much does it interfere with sex, life, or regular activities compared to other methods that you have tried?

19. **Which of the three study products (tablets, injections, and ring) did you like the most? Why?**

Possible probing topics:

- Which product is easiest to use? What makes it easy?
- What is the most desirable product feature?
- Would you choose to use this product instead of condoms to protect yourself against HIV and unplanned pregnancy, assuming it was just as effective?

20. **Which of the three study products (tablets, injections, and ring) did you like the least? Why?**

Possible probing topics:

- Which product is hardest to use? What makes it hard?
- What is the least desirable product feature?

Recommendations and Feasibility of Future Use

21. **Which one of the three study products (tablets, injections, or ring), if any, do you think your friends would be most likely to use in the future? Which one would you be most likely to use?**

Possible probing topics:

- What product features are most important to you and your friends?
- Do you think any of the products would not be used even if they were available?
- Do you think any of the products are likely to be stigmatized by the community?

22. **Which one of the three study products (tablets, injections, or ring) did you choose to use for the next part of the study?**

Possible probing topics:

- Why did you choose this one?
- What influenced your decision?
- Was it difficult to choose?
- At the start of the study would you have guessed that you would have chosen this one?
