# Supplementary material for: Female and male partner perspectives on placebo Multipurpose Prevention Technologies (MPTs) used by women in the TRIO study in South Africa and Kenya
Source: PLoS One. 2022 May 12;17(5):e0265303. doi: 10.1371/journal.pone.0265303 (PMC9097999; doi:10.1371/journal.pone.0265303)
Supplement: S4 File — IDI guide for second round female participant interviews. (PDF) [file pone.0265303.s004.pdf]

Trio Study

In-depth Interview (IDI) Topic Guide for Female Clinical Study Participants, Round 2 (Exit)

INSTRUCTIONS for the Interviewer: How to use the IDI Guide

1. There are two levels of questions:

   • Primary interview questions: appear in **bold** text. They address the topics that you as the interviewer must ask and discuss with participants. The questions are suggestions for getting the discussion going. You are not required to read them verbatim, but they are written to ensure some consistency across IDIs. You may adapt the questions and/or ask them in a different order, depending on how the interview develops. However you will have to ensure that by the end of the interview, all the topics and key themes have been covered.

   • Probing topics: are indicated with a bullet. If you find that the participant provides little information in response to the primary question, these probing topics may be used to encourage further discussion. You are not required to cover every topic listed. So, depending on what has already been discussed, and the IDI context, you may ask these probes or not.

2. *Instructions/suggestions to interviewer are in italics and [brackets].*

3. Words found in (parentheses) are meant to provide wording options to interviewers to fit various situations. For example, they often provide a present or past tense verb.

4. The IDI guide is not meant to be used to take notes. Rather, you should use the separate notes form, where you will also insert your initials, the participant's PTID, as well as the date, start and end time of the interview.

[*Start Recorder and Read Introduction*]: My name is _________________. Thank you again for your willingness to be a part of this discussion. I am looking forward to hearing your thoughts on the MPT product(s) you have been using. Please know there are no right or wrong answers and we welcome every opinion about the topics we will discuss, so feel free to share your thoughts, opinions, and views openly. If during our discussion, there are issues or concerns that you would like to talk about, feel free to bring them up, even if I didn't ask about them. If you have specific questions during the interview, I will take note of them and answer them directly after the interview. If I cannot answer them, I can refer you to someone who may be able to help.

The main goal of this discussion is to better understand your experience with the product(s) you chose to use in the second half of the Trio study. I want to remind you that what we discuss here will be kept confidential, and that we will not share your personal information or responses with anyone outside of the study. What you share will help scientists create products that women like you will actually want to use, so we especially want to hear if there was anything you didn't like about the products, so we can improve them.

Section A. Warm-up and Study Experience
**First we are going to talk about your experience being part of this study.**
1. **Tell me about your experience being part of Trio (*Do not discuss specific products with her).***   *Possible probing topics:*   • Experiences with introductory workshop   • Experiences with other engagement activities (phone check-ins, video, educational materials, certificate of achievement/honesty pledge, product counseling, comment box, retention workshops)   • Suggestions or ideas on how to make participants feel more engaged   • Challenges to participating (individual, partner, social, community level challenges)   • Experiences during study visits or at the clinic, including interactions with other participants and with study staff   • Effects of study participation on daily life for participant, partner(s) and others   • Changes in decision-making or communication with male partner(s), on topics such as contraception/pregnancy and HIV prevention
2. **How would you describe your role in Trio to a friend? (what did you do and why?)**   *Possible probing topics:*   • How the participant viewed the goal of the study   • Opinions about using only placebo products in a research study   • Whether it was helpful to try all three products to form an opinion about each of the products   • Whether it was helpful to try all three products before choosing one

Section B. Product Choice
**Now let's talk about the product you chose to use after trying the tablets, injections, and ring.…***(INTERVIEWER: SKIP TO Q. 3A IF PARTICIPANT REFUSED TO CHOOSE PRODUCT)*
3. **Tell me about the product(s) you chose to use and why you chose it (them).**   *Possible probing topics):*   • What the participant liked most about it (them), anything she really loved and that stood out as positive   • Positive / desirable features (taste, smell, size, color, texture, feel, discretion, perceived efficacy, regimen)   • What, if anything, the participant really disliked about it (them)   • Convenience/ease of use (how often product is used, how easy it is to obtain, how easy it is to use)   • How product compares to the other two study products   • Why and how did you select this product – decision-making process and influences   ***(GO TO QUESTION 4 ON NEXT PAGE)***

3A. (ASK IF PARTICIPANT REFUSED TO CHOOSE A PRODUCT) **Tell me about why you decided not to choose a product to use after you had a chance to try each one for a month.**

Possible probing topics (only ask if participant decided not to choose product):

- Why the participant decided not to choose and use a product for the rest of the study
- Negative/ undesirable features
  - Physical characteristics: size, feeling, shape, color, smell
  - Stigma associated with medication or HIV (perceived efficacy)
  - Comfort/discomfort using the product (e.g. depending on product – vaginal-insertion/removal/injection site/swallowing issues, duration, effect on sex, use during menses)
  - Ease of use (access to clinic, who administers the product - patient/clinician, convenience, where product can be accessed)
  - Privacy and storage issues (how discreet is the method)
- What, if anything, the participant liked about the products she did try in the first part of the study
- Why and how participant decided not to select one for the use period
  - Decision-making process and influences

(SKIP TO SECTION D ON PAGE 5)

4. **At your last visit you were given the option to switch products. How did you decide what to do?**

Possible probing topics:

- Positive and negative experiences with first product
- Decision-making process and influences
- Experiences with second product (if applicable)
- Experiences with using same product for 2 months (if no switch)
- Changes in experience and preference over time

5. **How was your experience using the product(s) in the last 2 months different from using them at the start of the study?**

Possible probing topics:

- Changes in comfort with, or acceptability of product
- Changes in how often product was used
- Change in other's opinion of the product (e.g. opinions of partner, family, friends, peer participants)

Section C. Product Adherence

Now we are going to talk about what it was like to use the product(s) you chose. You were asked to try to use your product consistently for the past two months. It is most important to us to understand whether you were really able to use them, and your reasons for doing so or not doing so. Only truthful information can help scientists to develop products women will actually want.

(DO NOT READ IF PARTICIPANT CHOSE INJECTIONS: **If you did not use the products, that is OK, we just want to know WHY.)**

6. **Tell me about your experience using the product(s) in the past 2 months.**

Possible probing topics:

- Ease of use
- Partner reactions

(Interviewer: If the participant chose injections, skip probes below)

- Adherence – how consistent was use
- Experiences with non-use
- Challenges to using consistently
- Impact of products being placebo on ability to use or not, consistently

7. **What sorts of things about the area you live in helped or hindered your ability to use the study product(s) as directed?**

Possible probing topics:

- Logistical issues (i.e. job flexibility, public transportation, having transport money in advance)
- Privacy and discreetness (i.e. carrying products, attending the clinic which is associated with HIV)
- The scheduling of clinic visits (time of day, day of the week)
- Dangerous places – e.g.: is the route to the clinic dangerous?

8. **What aspects of where you live/ your home made it easy or difficult to use the study product(s)?**

Possible probing topics:

- Storage and disposal of products (tablets only)
- Competing household obligations (daily duties/chores/caregiving to sick family members)
- Privacy

9. **How did the people who are close to you or who live with you influence your use of the study product(s)?**

Possible probing topics:

- Disclosure about study and products: to whom, what, and how – eg. 'voluntary' disclosure or accidental disclosure, motivation to disclose
- Relationship of these people to participant
- Role of other family or household members in sexual decision-making, use of product and condoms
- Support or lack of support from these people
- Help from others in the form of reminders to use the study products

10. **If you have a primary partner, how did he influence your use of the study product(s)?**

Possible probing topics:

- Disclosure to partner regarding product use and the consequences of this
- Any conflicts between participant and her partner that were generated or intensified by the study products (What type of conflicts? Explain when and how)
- Support or lack of support from partner (e.g., reminding you to take product)

11. **What was the most challenging part about using the study product(s) consistently?**

Possible probing topics:

- Willingness and ability to use
- Concerns about use
- Motivations and interest to use
- No active ingredients/medicines
- Remembering to use *(N/A if chose injections)*

12. **How would you change the study product(s) you chose to make it easier to use?**

Possible probing topics:

- Changes to the physical features (size, texture, etc.)
- Changes to the frequency of use (daily, monthly, etc.)
- Changes to who administers it (you vs clinician)
- Changes to where you can get it (clinic, pharmacy, community center, other)
- Any other types of changes that would make products easier to use

Ask these questions only if the participant chose the ***tablets****:*
13. **Tell me about the last time you did not use the tablets.**<br>*Possible probing topics:*<br>• What was going on<br>• Detailed circumstances: where, when, how, who was there, what happened<br>• Frequency of these circumstances<br>• Reasons why tablets were not used last time, but they were used another time

Ask these questions only if the participant chose the ***ring****:*
14. **Tell me about the times when you took the ring out.**<br>*Possible probing topics:*<br>• How often the ring was out<br>• Reasons the ring was out:<br>o Took it out for sex<br>o Took it out during menses<br>o Felt uncomfortable (physical discomfort as well as emotional discomfort)<br>o Concerns about hygiene or other worries about the ring<br>o Moved out of place or came out
15. **Tell me about the last time when you took the ring out or it came out on its own, either partially or fully.**<br>*Possible probing topics:*<br>• Timing and circumstances when ring was removed or came out<br>• Instances of partner removing ring<br>• Position of her body when ring came out<br>• What did she do about it<br>• [*If re-inserted outside of the clinic*] was ring cleaned and how

Section D. Product Preference and Future Use
Now we're going to talk about which products you would prefer to use in the future.
• **<u>If you were not planning to get pregnant</u>, would you prefer to use a product that protects against both HIV and pregnancy, or would you rather use two different products – one for HIV prevention, and another for pregnancy prevention?**<br>*Possible probing topics:*<br>• Advantages and disadvantages of using a single product vs two separate products<br>• Any worries about using a new medical product for both pregnancy and HIV prevention<br>• Ways a 2 in 1 (multipurpose) product makes taking care of health easier/harder<br>• Whether participant would perceive a single vs. a multipurpose product to impact her health differently (positively or negatively)<br>***Interviewer: Show Copackaged Pills Visual Aid and ask for opinions and comparisons to single tablet***<br>***a) Photo 1: The first photo shows two pills—one for HIV prevention and one for pregnancy prevention—that are packaged together, but as separate pills.***<br>***b) Photo 2: These pills would be swallowed one at a time, both daily.***<br>***c) Photo 3: This is an alternative to using a single tablet for both purposes, shown here.***

16. **Assuming they were equally protective, if you could use one of the three study products (tablets, injections, ring) or condoms to prevent both pregnancy and HIV in the future, what method would you choose and why?**

Possible probing topics:

- Positive features of that product
- How product is administered
- Duration of use
- How disruptive or discreet it is
- How much it interferes with sex, life or regular activities compared to other methods
- How effective you perceive it to be

17. **Are there other MPT products you would prefer to the ones you tried in TRIO?**

Possible probing topics:

- Explore other delivery forms: implants, other vaginal formulations ***(Interviewer: show other products using Other MPT Products Visual Discussion Tool and ask for opinions on other products)***
- Explore other product durations: e.g. injections or rings that last 2 or 3 months/ ideal duration
- Explore who administers the product (self-administered vs provider administered)

Section E. Messaging

18. **What would be the best way to inform and educate women about new multipurpose products?**

Possible probing topics:

- Advertisements in clinics
- Newspapers, television, billboards, radio
- Community meetings
- Information that should be included in the advertisements
- Ideas for how the advertisements should look
- Similarities and differences with existing messages about condoms
- Ways to communicate this information to women, to their male partners or family members
- Where and from whom should people receive the education about MPT
- Similarities and differences in messages about MPT products depending on whether they are tablets, rings or injections

(Ask these additional messaging questions if there is sufficient time)

19. **How can scientists make these new products more attractive to young women? What strategies should be used?**

Possible probing topics:

- Packaging
- Messaging/delivery mechanism
- Locations/access
- Suggestions for other ways to get input from women on new products

20. **Once the product is developed, what do you think would be the best way to inform women that this product is available?**

Possible probing topics:

- Advertisements in clinics?
- Newspapers, television, billboards, radio?
- Community meetings?
- What are the most trusted sources for information about a product like this?
- What would the advertisement look like? What would information would it include?

21. **If these products from Trio were proven effective and made available with active ingredients in them, how would you promote them to your sisters and friends?** ***(Interviewer: be clear on WHICH of the products the participant is referring to with this description.)***

Possible probing topics:

- What would you say is the main reason to use it?
- What's the benefit of using one of these multipurpose products vs. condoms or other things you can do to prevent HIV and pregnancy?
- What features would you highlight and which features would not be good for promotion purposes?
- Are there features or aspects you would need to explain because you think they would stop your friends from using?
- What would you call such a multipurpose product? (e.g. a wellness product, a sexual health product, women's health product, a 2-in-1)?
- Would the names be different for oral tablets vs a vaginal ring vs injections? For example, if the name were Trust – could it be Trust oral tablets, Trust vaginal ring, etc.?
