# Supplementary material for: Female and male partner perspectives on placebo Multipurpose Prevention Technologies (MPTs) used by women in the TRIO study in South Africa and Kenya
Source: PLoS One. 2022 May 12;17(5):e0265303. doi: 10.1371/journal.pone.0265303 (PMC9097999; doi:10.1371/journal.pone.0265303)
Supplement: S5 File — IDI guide for male partner interviews. (PDF) [file pone.0265303.s005.pdf]

Trio

Male Partner In-depth Interview (IDI) Topic Guide

INSTRUCTIONS for the Interviewer: How to use the IDI Guide

1. There are two levels of questions:

   • Primary interview questions: appear in **bold** text. They address the topics that you as the interviewer must ask and discuss with participants. The questions are suggestions for getting the discussion going. You are not required to read them verbatim, but they are written to ensure some consistency across IDIs. You may adapt the questions and/or ask them in a different order, depending on how the interview develops. However you will have to ensure that by the end of the interview, all the topics and key themes have been covered.

   • Probing questions: are indicated with a bullet. If you find that the participant provides little information in response to the primary question, these questions may be used to encourage further discussion. You are not required to ask every question listed. So, depending on what has already been discussed, and the IDI context, you may ask these probes or not.

2. *Instructions/suggestions to interviewer are in italics and [brackets].*

3. Words found in (parentheses) are meant to provide wording options to interviewers to fit various situations. For example, they often provide a present or past tense verb.

4. The IDI guide is not meant to be used to take notes. Rather, you should use the separate notes form, where you will also include your initials, the participant's PTID, as well as the date, start and end time of the interview.

Before starting the IDI, ensure the participant has provided written informed consent and demographic information.

[***Start Recorder and Read Introduction***]: My name is _________________. Thank you again for your willingness to be a part of this discussion. I am looking forward to hearing your thoughts on the products your partner used during the Trio study. Please know there are no right or wrong answers and we welcome every opinion about the topics we will discuss, so feel free to share your thoughts, opinions, and views openly. If, during our discussion, there are issues or concerns that you would like to talk about, feel free to bring them up, even if I didn't ask about them. If you have specific questions during the interview, I will take note of them and answer them after the interview. If I cannot answer them, I can refer you to someone who may be able to help. Before we start, can you confirm for me that you have already provided written informed consent to take part in this discussion? [*Wait for oral confirmation to begin*].

The main goal of this discussion is to better understand your experience with the products your partner used in the Trio study. I want to remind you that what we discuss here will be kept confidential, and that we will not share your personal information or responses with your partner or anyone outside the study.

Background and relationship information with Trio participant
First we are going to talk about you and your relationship with *XX [female partner from Trio]*.
1. **Tell me about you, your partner, and your relationship?**<br>*Possible probing questions:*<br>• Where is your family from, how long have you lived in this area?<br>• Do you have children? Are you planning to have (more) children? When?<br>• How and when did you meet your partner *[Female partner from Trio]*?<br>• What is your relationship like? (marital status, living together, etc.)<br>•<br>2. **How are decisions made with your partner(s) about sex and prevention?**<br>• What kind of discussions do you have with your partner about family planning? Who makes the decisions about what family planning method to use?<br>• What kind of discussions do you have about HIV? …and about preventing HIV?<br>• Tell me how you and your partner discuss sex, for example whether to have sex, whether to use a condom.<br>• Tell me about other sex partners besides *[female partner from Trio]* – how are those partner(s) and relationships different than this one?

Trio Knowledge
Now, we are going to talk about what you know about the Trio study.
3. **What do you think the Trio study was about?**<br>*Possible probing questions:*<br>• What did your partner tell you about the study and the study products?<br>• When did she tell you she had joined the study?<br>•<br>• What products did she try?<br>• Which product did she choose to use for the last month and why?<br>• What did you understand about how each of the study products "worked" (e.g., how often they had to be used/taken, how they were used, where the ring is placed, etc.)?<br>• Did you learn about the Trio study any other way? (clinic staff, internet, friends, etc.)<br>• Are you aware of any opinions, conversation about the Trio study in the community?

Demonstration of Trio Products
(Interviewer read) I am now going to show you the three different products that your partner used during the Trio study. These are being developed for women to use to prevent HIV and unplanned pregnancy. The whole purpose of Trio is to see what women like best. *(Interviewer: place sample products on table)*<br><br>This one is a ring that a woman inserts into her vagina and leaves it there for one month. It is designed to slowly release medicines to prevent HIV and unplanned pregnancy.<br><br>These are tablets, or pills. For these to work a woman would have to swallow one every day.<br><br>The third product is an injection. Women would have to go to the clinic and receive one injection in each buttock muscle – so two shots – every month.
4. **Do you have any questions for me before we talk more about these products?**

Product Acceptability
5. **What was your opinion of the study products your partner used?**<br>*Possible probing questions:*<br>• How much did you like the tablets (explain the positives and negatives)?<br>• How much did you like the injections (explain the positives and negatives)?<br>• How much did you like the ring (explain the positives and negatives)?<br>• What was your reaction when you first heard about the study products? How did your opinion change over time?
6. **What effect did the ring have on sex with your partner(s)?**<br>*Possible probing questions:*<br>• How did the ring affect sex with your partner?<br>• In what ways did it help or hinder your sexual relationship(s)?<br>• Are there any ways in which a change in the ring size or the way it feels could improve your sexual experience?<br>• How did the tablets or the injection have an impact on sex?

Male Partner Involvement
7. **In what ways did you influence your partner's use of the tablets, injections, or ring?**<br>*Possible probing questions:*<br>• Tell me how you were involved in and influenced her use of each of the products… (probe individually to cover tablets, ring and injection)<br>• Did using products create problems between you and your partner? How? (What type of problem? Explain when and how.)<br>• Did the study change how you discussed sex with your partner? How? HIV? Family Planning? Other?

Product Preference
8. **Did you prefer for your partner to be on the tablets, injections, or ring?**<br>*Possible probing questions:*<br>• Which of the study products did you like the least and most and why?<br>• What are the advantages/disadvantages of using a daily tablet versus a monthly ring or injections every two month?<br>• Which product(s) do you think other men would prefer that women use to prevent both pregnancy and HIV?<br>• Which ones do you think other men you know really wouldn't like? Why?
9. **How do you feel about your partner using a multipurpose product to prevent HIV and pregnancy compared to you wearing a condom?**<br>*Possible probing questions:*<br>• What are the benefits of men having control over an HIV and pregnancy prevention product (like the condom)?<br>• And what problems might there be if the man has control?<br>• What are the benefits of women having control over an HIV and pregnancy prevention product (like the ones in this study)?<br>• And what are potential problems if the woman has control?<br>• What other factors influence your opinion about whether you prefer a condom or a multipurpose product?<br>• In what situations would you <u>not</u> want your partner to use a multipurpose product?<br>• Is a multipurpose product suitable for all types of women and relationships (e.g. unmarried women, married women, casual partners)? For what types of relationships/couples do you think this is the right product?
10. **Are there other multipurpose products you would prefer your partner to use, compared to the ones she tried in Trio?**<br>*Possible probing questions:*<br>• How does men's preference for tablets or ring or injection depend on (or relate to) the type of partner (e.g. casual, dating/unmarried, married)?<br>• *Explore other delivery forms: implants, co-packaged pills, and other vaginal formulations (Interviewer: show other products using Other MPT Products Visual Discussion Tool)*<br>• *Explore other product durations: e.g. injections or rings that last 2 or 3 months/ ideal interval?*

Male Involvement
11. **How important is it for researchers to interact directly with men and women in this community as they are developing new multipurpose technology products?**<br>*Possible probing questions:*<br>• In past research, we have tried to involve men in research and it has been hard to get them interested in participating. What suggestions do you have for better ways to engage men?<br>• Why is it important to involve men in this kind of research?<br>• Why did you agree to participate in this interview?

- How should men support their female partners? Is it different if it is your wife vs. your girlfriend vs. a casual partner?
- What suggestions do you have for how we can work with men who do not want their female partner participating in research?
- What role can men play in helping connect researchers to the larger community?
- What suggestions do you have for how researchers can engage women in research?
- How about the larger community?
- How should other men in the community be involved?
- What will happen if men are not involved?

Messaging

12. **Although these multipurpose products are designed to be used by women, men may want to know about them. What would be the best way to reach men with information about new multipurpose products for women?**

Possible probing questions:

- Should they be promoted and explained similarly or differently from condoms?
- How should this information be communicated, to men and to women?
- How should the information be different for men versus women?
- How about information and messages for couples?
- Where and from whom should people receive the education about MPT?
- Should the information and messages be similar or different, depending if the new MPT products are tablets, rings or injections?
